# Association between Soluble (Pro)Renin Receptor Concentration in Cord Blood and Small for Gestational Age Birth: A Cross-Sectional Study

**DOI:** 10.1371/journal.pone.0060036

**Published:** 2013-03-21

**Authors:** Noriyoshi Watanabe, Satoshi Morimoto, Takeo Fujiwara, Tomo Suzuki, Kosuke Taniguchi, Takashi Ando, Tadashi Kimura, Haruhiko Sago, Atsuhiro Ichihara

**Affiliations:** 1 Department of Endocrinology and Hypertension, Tokyo Women's Medical University, Shinjuku, Tokyo, Japan; 2 Department of Maternal-Fetal and Neonatal Medicine, National Center for Child Health and Development, Setagaya, Tokyo, Japan; 3 Department of Social Medicine, National Research Institute for Child Health and Development, Setagaya, Tokyo, Japan; 4 Department of Obstetrics and Gynecology, Osaka University, Suita, Osaka, Japan; Baylor College of Medicine, United States of America

## Abstract

**Objective:**

The (pro)renin receptor [(P)RR] has been recognized as a multifunctional receptor. The purpose of this study was to assess the association between plasma soluble (P)RR [s(P)RR] concentration in human cord blood (i.e., neonatal blood at birth) and small for gestational age (SGA) birth.

**Methods:**

Participants were women with a singleton pregnancy who delivered at the National Center for Child Health and Development between January 2010 and December 2011. Inclusion criteria were availability of maternal pre-pregnancy and paternal body mass index, and the absence of structural anomalies in neonates. s(P)RR concentration in cord blood was measured in 621 neonates. The 621 pairs of mothers and neonates were categorized into four groups based on quartiles of s(P)RR concentrations in cord blood. SGA was defined as a birth weight below the 10^th^ percentile for gestational age. Logistic regression analysis was performed to assess the association between cord plasma s(P)RR concentration (quartiles) and incidence of SGA births.

**Results:**

Among 621 neonates, 55 (8.9%) were diagnosed as SGA (SGA group) and 566 (91.1%) were not (non-SGA group). Average s(P)RR concentration in cord blood was 66.1±12.6 ng/ml (mean±standard deviation). There were 155 pairs in the first plasma s(P)RR concentration quartile (Q1: <58.2 ng/ml), 153 pairs in the second quartile (Q2: 58.2–65.1 ng/ml), 157 pairs in the third quartile (Q3: 65.1–73.1 ng/ml) and 156 pairs in the fourth quartile (Q4: >73.1 ng/ml). The distribution of SGA births was 18 (11.6%) in Q1, 14 (9.2%) in Q2, 16 (10.2%) in Q3 and 7 (4.5%) in Q4, respectively. The odds ratio of SGA births was 0.24 (95% confidence interval: 0.08–0.71) for the fourth quartile compared to the first quartile in multivariate models. The *P*-value for trend was also significant (*P* = 0.020).

**Conclusion:**

High s(P)RR concentration is associated with a lower SGA birth likelihood.

## Introduction

The (pro)renin receptor [(P)RR] was initially thought to contribute to the renin-angiotensin system (RAS) [1]. Recent studies, however, have revealed (P)RR to be a multifunctional receptor. In addition to regulating the RAS, (P)RR is closely associated with the vacuolar-type H+-ATPase (V-ATPase), which is important for the maintenance of intracellular pH [2]–[3], and Wnt signaling, an important component of embryonic development [4]–[6]. While *in vitro* studies have revealed a role for (P)RR in fetal development, no studies have addressed the association between (P)RR and fetal development *in vivo*.

(P)RR exists in three different molecular forms: 1) a full-length integral transmembrane protein, 2) soluble (P)RR [s(P)RR] in plasma and urine and 3) a truncated form that includes only the transmembrane and cytoplasmic domains [7]. Of these, only s(P)RR can be measured in a blood sample. Recently, we developed an s(P)RR enzyme-linked immunosorbent assay (ELISA) kit [8] that allows s(P)RR quantitation in clinical settings.

Small for gestational age (SGA) infants are diagnosed when birth weight falls in the 10^th^ percentile at each gestational age, and are reported to be at an increased risk of perinatal morbidity and mortality [9]–[12]. Moreover, SGA infants have reduced cardiovascular, renal and metabolic function [13]. In two recent systematic reviews, SGA infants delivered at term were also reportedly at risk of neurodevelopmental delay [14]–[15]. Despite the fact that SGA is recognized as a risk factor in both perinatal and postnatal stages, little is known about its pathophysiology.

In the present study, we hypothesized that (P)RR contributed to SGA birth prevalence. To confirm our hypothesis, we conducted a cross-sectional study to assess the association between plasma s(P)RR concentration in human cord blood (i.e., neonatal blood at birth) and SGA.

## Materials and Methods

### Study participants

This study was approved by the Ethics Committee of the National Center for Child Health and Development (NCCHD). The study included Japanese women who delivered at the NCCHD hospital between January 2010 and December 2011, and their neonates. Inclusion criteria were a singleton pregnancy, availability of maternal pre-pregnancy and paternal body mass index (BMI) data and absence of structural anomalies in the neonates. We enrolled and obtained written informed consent from all participants during pregnancy (before labor pain onset). All blood samples were obtained from the umbilical vein at delivery. We weighed all neonates just after birth using neonatal digital scales (UDS-200Be-K, Yamato Scale Co. Ltd., Hyogo, Japan). A birth weight below the 10^th^ percentile for gestational age was classified as SGA. Birth weight for gestational age was determined using the percentile scale derived from the formula used in Japan [16]. Data for maternal characteristics and paternal BMI were available from our perinatal database and medical records. Although we initially enrolled 713 women in the study, we excluded 92 women for whom we could not obtain enough blood for analysis, resulting in the analysis of 621 pairs of mothers and neonates. Among 621 neonates, 55 (8.9%) were diagnosed as SGA (SGA group) and 566 (91.1%) were not (non-SGA group).

### Quantitation of s(P)RR

Assays were performed by someone who was blinded to the outcome of the pregnancy. Enzyme-linked immunosorbent assays (ELISAs) for human s(P)RR were performed in duplicate, as previously described, using commercial kits (Immuno-Biological Laboratories Co., Fujioka, Japan) [8]. Plasma samples were used to measure s(P)RR concentrations. The detection limit for the s(P)RR assay for this particular ELISA kit was 24 pg/ml, with inter-assay and intra-assay coefficients of variation of 7.5% and 5.5%, respectively.

### Statistical analysis

Differences in baseline characteristics and birth outcomes between SGA and non-SGA groups were compared using the chi-square test or Fisher's exact test for categorical variables, and the t-test or Wilcoxon rank sum test for continuous variables.

To examine the association between baseline characteristics, demographics, or birth outcome, and s(P)RR concentration, participants were categorized into four groups based on s(P)RR concentration in cord blood (i.e., quartiles). Associations between s(P)RR quartiles and baseline characteristics or birth outcome were assessed by regression analysis for continuous variables and logistic regression for categorical variables.

Logistic regression analysis was used to investigate the association between cord plasma s(P)RR concentration quartile and SGA. All multivariate models were adjusted for variables if p values were below 0.2 in the aforementioned analyses (i.e., comparison of SGA and non-SGA groups, and the association between s(P)RR quartiles and baseline characteristics or birth outcome). We performed two sub-analyses to assess sensitivity, which excluded participants with 1) complications (e.g., hypertension), and 2) gestational age <39 weeks, as these factors might be associated with s(P)RR levels [17] and SGA [18]–[19]. All analyses were performed using STATA software (version 12.0; Stata Corporation, College Station, TX).

## Results

### Distribution of s(P)RR concentration in umbilical cord blood

**Figure 1:**
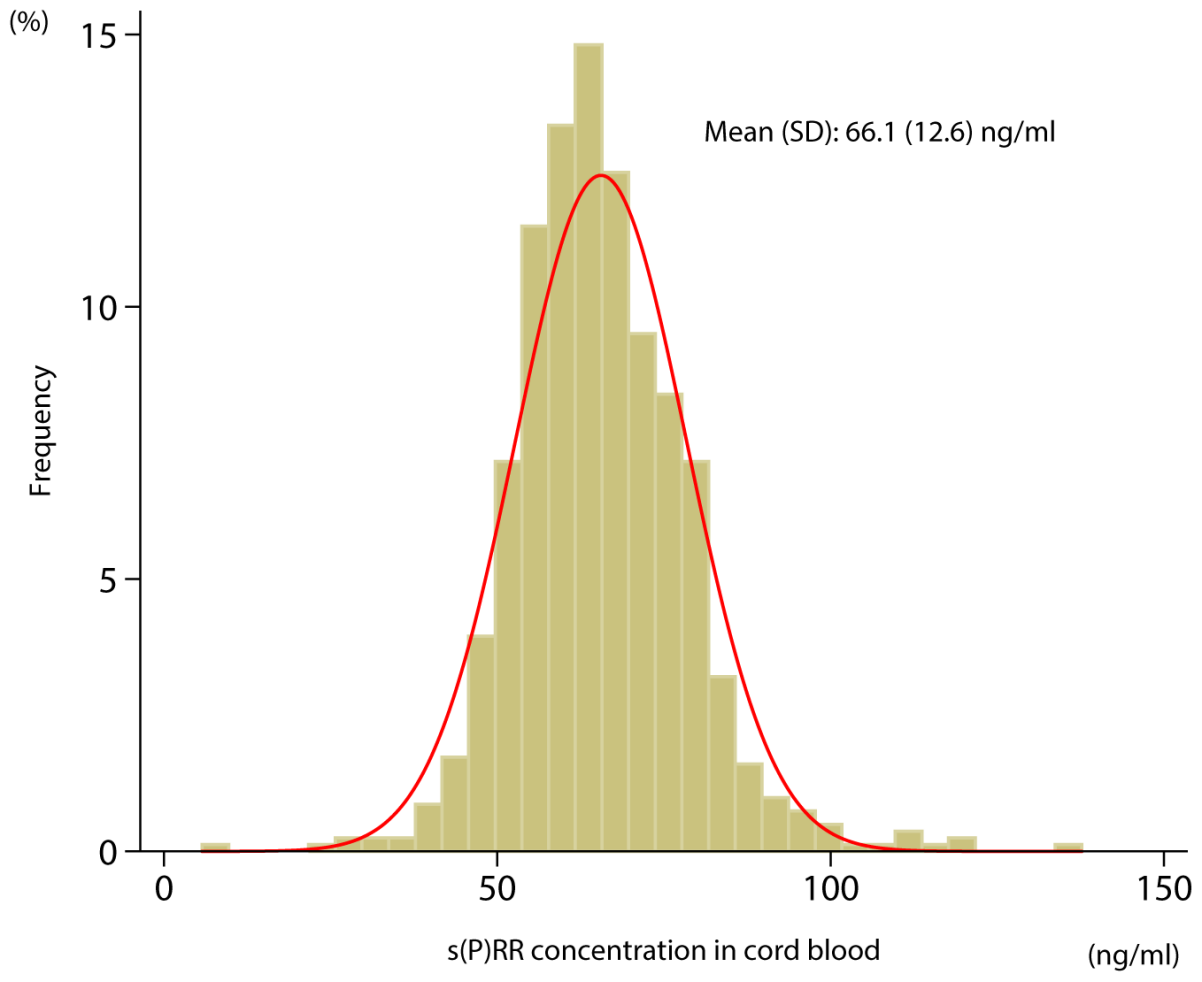
Frequency histogram of plasma soluble (pro)renin receptor [s(P)RR)] concentration in cord blood. Data are shown as proportions of s(P)RR concentrations measured in 4 ng/ml increments.

Figure 1 shows that s(P)RR concentration in umbilical cord blood was normally distributed. The mean s(P)RR concentration was 66.1±12.6 ng/ml (mean±SD), and concentrations ranged from 26.9 to 136.8 ng/ml.

### Comparison of baseline characteristics and birth outcomes between SGA and non-SGA groups

A comparison of baseline characteristics and birth outcomes between SGA and non-SGA groups are shown in Table 1. Prevalence of women who conceived by *in vitro* fertilization was lower in the SGA group than in the non-SGA group (3.5% vs. 16.4%, p = 0.031). Gestational weight gain was smaller in the SGA group than in the non-SGA group (8.3 kg vs. 9.4 kg, p = 0.037). Birth weight and placental weight were both lower in the SGA group than in the non-SGA group (2367 g vs. 3032 g, p<0.001, and 431 g and 545 g, p<0.001, respectively). Prevalence of hypertensive disorder was higher in the SGA group than in the non-SGA group (16.4% vs. 6.5%, p = 0.014). Finally, s(P)RR concentration in cord blood was lower in the SGA group than in the non-SGA group (62.9 ng/ml vs. 66.6 ng/ml, p = 0.049).

**Table 1:** Comparison of baseline characteristics and birth outcomes between SGA and non-SGA groups.

	Total (n = 621)	SGA (n = 55)	non-SGA (n = 566)	P value
**Baseline characteristics**				
Age (yrs), mean (SD)	35.6 (4.2)	34.8 (4.4)	35.6 (4.1)	0.17
Primiparous [no. (%)]	353 (56.8)	32 (58.2)	321 (56.7)	0.83
Pre-pregnancy BMI (kg/m^2^), mean (SD)	20.6 (3.1)	20.2 (2.4)	20.6 (3.2)	0.33
Paternal BMI (kg/m^2^), mean (SD)	23.4 (2.9)	22.9 (2.6)	23.4 (2.9)	0.25
Smoking during current pregnancy, [no. (%)]	20 (3.2)	1 (1.8)	19 (3.4)	>0.99
Drinking during current pregnancy, [no. (%)]	13 (2.1)	0 (0)	13 (2.3)	0.62
Family history of diabetes mellitus, [no. (%)]	72 (11.6)	7 (12.7)	65 (11.5)	0.83
Family history of hypertension, [no. (%)]	107 (17.2)	11 (20.0)	96 (17.0)	0.58
Conceived by in vitro fertilization, [no. (%)]	96 (15.5)	3 (5.5)	93 (16.4)	**0.031**
**Pre-existing maternal complications**				
Hypertension, [no. (%)]	17 (2.7)	1 (1.8)	16 (2.8)	>0.99
Diabetes mellitus, [no. (%)]	1 (0.2)	0 (0.0)	1 (0.2)	>0.99
Asthma, [no. (%)]	4 (0.6)	0 (0.0)	4 (0.7)	>0.99
Renal disease, [no. (%)]	1 (0.2)	0 (0.0)	1 (0.2)	>0.99
Hyperthyroidism, [no. (%)]	5 (0.8)	0 (0.0)	5 (0.9)	>0.99
Hypothyroidism, [no. (%)]	8 (1.3)	0 (0.0)	8 (1.4)	>0.99
Autoimmune disease, [no. (%)]	10 (1.6)	1 (1.8)	9 (1.6)	0.61
**Birth outcomes**				
Gestational age at delivery (wks), mean (SD)	39.1 (1.6)	38.8 (2.8)	39.2 (1.5)	0.09
Gestational weight gain (kg), mean (SD)	9.3 (3.7)	8.3 (4.2)	9.4 (3.6)	**0.037**
Neonatal sex, [no. female (%)]	295 (47.5)	28 (50.9)	267 (47.2)	0.67
Birth weight (g), mean (SD)	2973 (420)	2367 (500)	3032 (361)	**<0.001**
Placental weight (g), mean (SD)	535 (103)	431 (100)	545 (97.7)	**<0.001**
Hypertensive disorders in pregnancy*, [no. (%)]	46 (7.4)	9 (16.4)	37 (6.5)	**0.014**
Gestational diabetes, [no. (%)]	41 (6.6)	3 (5.5)	38 (6.7)	>0.99
s(P)RR concentration in cord blood (ng/ml), mean (SD)	66.2 (13.4)	62.9 (11.9)	66.6 (13.4)	**0.049**

SGA denotes small for gestational age, SD: standard deviation, BMI: body mass index, s(P)RR: soluble (pro)renin receptor

* Hypertensive disorders in pregnancy include gestational hypertension, pre-eclampsia, chronic hypertension and superimposed hypertension

### Association between baseline characteristics, demographics, or birth outcome, and s(P)RR concentration in cord blood

Table 2 shows associations between baseline characteristics, demographics, or birth outcomes, and s(P)RR concentration in cord blood. There were 155 pairs in the first plasma s(P)RR concentration quartile (Q1: <58.2 ng/ml), 153 pairs in the second quartile (Q2: 58.2–65.1 ng/ml), 157 pairs in the third quartile (Q3: 65.1–73.1 ng/ml) and 156 pairs in the fourth quartile (Q4: >73.1 ng/ml). Gestational age at delivery in Q4 was somewhat earlier than in the other three quartiles by multiple comparison with Bonferroni correction (Q1 vs. Q4: *P*<0.001; Q2 vs. Q4: *P* = 0.002 and Q3 vs. Q4: *P* = 0.009). There were no significant differences in other baseline characteristics and birth outcomes among the four quartiles.

**Table 2:** Association between baseline characteristics, demographics, or birth outcome, and s(P)RR concentration in cord blood.

	Q1 (n = 155)	Q2 (n = 153)	Q3 (n = 157)	Q4 (n = 156)	*P* for trend
**Baseline characteristics**					
Age (yrs), mean (SD)	35.2 (4.0)	35.7 (4.3)	35.6 (4.3)	35.7 (4.2)	0.45
Primiparous [no. (%)]	94 (60.7)	78 (51.0)	95 (60.5)	86 (55.1)	0.69
Pre-pregnancy BMI (kg/m^2^), mean (SD)	20.5 (3.2)	20.8 (3.2)	20.5 (3.0)	20.7 (3.0)	0.74
Paternal BMI (kg/m^2^), mean (SD)	23.5 (3.0)	23.0 (2.6)	23.1 (2.3)	23.8 (3.4)	0.28
Smoking during current pregnancy, [no. (%)]	6 (3.9)	7 (4.6)	3 (1.9)	4 (2.6)	0.30
Drinking during current pregnancy, [no. (%)]	5 (3.2)	4 (2.6)	2 (1.3)	2 (1.3)	0.17
Family history of diabetes mellitus, [no. (%)]	23 (14.8)	16 (10.5)	21 (13.4)	12 (7.7)	0.11
Family history of hypertension, [no. (%)]	26 (16.8)	26 (17.0)	30 (19.1)	25 (16.0)	0.99
Conceived by in vitro fertilization, [no. (%)]	18 (11.6)	28 (18.3)	23 (14.7)	27 (17.3)	0.30
**Pre-existing maternal complications**					
Hypertension, [no. (%)]	4 (2.6)	6 (3.9)	4 (2.6)	3 (1.9)	0.14
Diabetes mellitus, [no. (%)]	0 (0.0)	0 (0.0)	0 (0.0)	1 (0.6)	NA
Asthma, [no. (%)]	0 (0.0)	0 (0.0)	2 (1.3)	2 (1.3)	0.12
Renal disease, [no. (%)]	0 (0.0)	0 (0.0)	0 (0.0)	1 (0.6)	NA
Hyperthyroidism, [no. (%)]	1 (0.7)	2 (1.3)	0 (0.0)	2 (1.3)	0.85
Hypothyroidism, [no. (%)]	3 (1.9)	1 (0.7)	2 (1.3)	2 (1.3)	0.74
Autoimmune disease, [no. (%)]	2 (1.3)	3 (2.0)	2 (1.3)	3 (1.9)	0.79
**Birth outcomes**					
Gestational age at delivery (wks), mean (SD)	39.4 (1.2)	39.3 (1.2)	39.2 (1.5)	38.6 (2.2)	<0.001
Gestational weight gain (kg), mean (SD)	9.1 (3.7)	9.5 (3.7)	9.1 (3.5)	9.5 (4.0)	0.65
Neonatal sex, [no. female (%)]	77 (49.7)	82 (53.6)	70 (44.6)	66 (42.3)	0.083
Birth weight (g), mean (SD)	3002 (383)	2975 (358)	2978 (431)	2936 (494)	0.19
Placental weight (g), mean (SD)	534 (95)	532 (99)	534 (98)	539 (118)	0.62
Hypertensive disorders in pregnancy*, [no. (%)]	9 (5.8)	10 (6.5)	15 (9.6)	12 (7.7)	0.36
Gestational diabetes, [no. (%)]	6 (3.9)	8 (5.2)	14 (8.9)	13 (8.3)	0.058

s(P)RR: soluble (pro)renin receptor.

First quartile of s(P)RR concentrations in cord blood (Q1): <58.2 ng/ml, second quartile (Q2): 58.2–65.1 ng/ml, third quartile (Q3): 65.1–73.1 ng/ml, fourth quartile (Q4): >73.1 ng/ml.

SD: standard deviation, BMI: body mass index, NA: not applicable.

* Hypertensive disorders in pregnancy include gestational hypertension, preeclampsia, chronic hypertension and superimposed hypertension.

### Distribution of SGA by s(P)RR quartile and association between s(P)RR concentration in cord blood and probability of SGA

Table 3 shows the distribution of SGA by cord plasma s(P)RR quartile and the results of logistic regression analysis of the association between cord blood s(P)RR concentration and the probability of SGA. The distribution of SGA was 18 (11.6%) in Q1, 14 (9.2%) in Q2, 16 (10.2%) in Q3 and 7 (4.5%) in Q4, respectively.

**Table 3:** Logistic regression analysis of quartiles of s(P)RR concentration in cord blood to determine the likelihood of SGA.

			Crude			Adjusted*		
	n	SGA, n (%)	OR	95% CI	p value	OR	95% CI	p value
Quartile 1 (<58.2 ng/ml)	155	18 (11.6)	ref	-	-	ref	-	-
Quartile 2 (58.2–65.1 ng/ml)	153	14 (9.2)	0.77	0.37 – 1.60	0.48	0.75	0.33 – 1.70	0.49
Quartile 3 (65.1–73.1 ng/ml)	157	16 (10.2)	0.86	0.42 – 1.76	0.69	0.79	0.35 – 1.78	0.57
Quartile 4 (>73.1 ng/ml)	156	7 (4.5)	0.36	0.14 – 0.88	0.026	0.24	0.08 – 0.71	0.010
P for trend			0.048			0.020		

s(P)RR denotes soluble (pro)renin receptor, SGA: small for gestational age, OR: odds ratio, CI: confidence interval

* All multivariate models were adjusted for maternal age, conception by in vitro fertilization, drinking during current pregnancy, family history of diabetes mellitus, preexisting hypertension, preexisting asthma, gestational age at delivery, gestational weight gain, hypertensive disorders during pregnancy, gestational diabetes, placental weight and neonatal sex

In the unadjusted model, the odds ratio of SGA was 0.36 for Q4 compared to Q1 (95% confidence interval [CI]: 0.14–0.88, *P* = 0.026). The *P*-value for trend was significant (*P* = 0.048). This association was also significant in multivariate models. The odds ratio of SGA was 0.24 for Q4 compared to Q1 (95% CI: 0.08–0.71, *P* = 0.010). The *P*-value for trend was also significant (*P* = 0.020). That is, neonates with a higher plasma s(P)RR concentration in cord blood were unlikely to be SGA.

### Sensitivity Analysis

First, we performed a logistic regression analysis that excluded women with any medical complications (hypothyroidism, hyperthyroidism, asthma, heart disease, collagen disease, preexisting hypertension and diabetes), gestational diabetes mellitus and hypertensive disorders in pregnancy. This revealed an SGA odds ratio of 0.16 (95% CI: 0.043–0.60) for Q4 compared to Q1, which was similar to the point estimate for the whole study population.

Second, we performed a logistic regression analysis excluding women who delivered at 39 weeks of gestation or earlier, and found an SGA odds ratio of 0.27 (95% CI: 0.91–1.10) for Q4 compared to Q1, which was also similar to the point estimate for the whole study population.

## Discussion

To the best of our knowledge, this is the first study to demonstrate the association between cord plasma s(P)RR concentration and fetal growth in humans. Specifically, neonates in the highest s(P)RR concentration quartile (Q4) were 0.24-fold less likely to be SGA than neonates in the lowest quartile (Q1), indicating that s(P)RR might be associated with appropriate fetal growth.

In the present study, s(P)RR concentrations in cord blood (i.e., neonates) were markedly higher than reported for normal adult volunteers [8] and pregnant women [17] in two previous studies. Higher s(P)RR levels in neonates compared to adults suggest that (P)RR may be essential for embryogenesis [20]. Recently, (P)RR has been recognized as a multifunctional receptor associated with the RAS, V-ATPase activity and Wnt signaling. (P)RR was initially characterized as a RAS component that contributes to the pathogenesis of cardiovascular disease [21]. Indeed, we demonstrated that elevated plasma s(P)RR early in pregnancy was significantly predictive of elevated blood pressure later in pregnancy, suggesting that (P)RR contributes to the tissue RAS during human pregnancy [17].

Among the three known functions of (P)RR (contribute to RAS, V-ATPase and WNT signaling), we surmise that the relationship between (P)RR and WNT signaling is highly significant, although the role of (P)RR in embryonic and fetal development remains unclear. Recent studies have identified (P)RR as an essential adaptor protein in the WNT signaling pathway [4], and have shown that WNT signaling plays a critical role in embryonic and fetal development [22]–[23]. Given that higher s(P)RR concentrations were associated with a lower SGA birth prevalence, (P)RR may function in normal embryonic and fetal development as an adaptor protein of WNT signaling.

Interestingly, a previous study found that higher maternal s(P)RR concentrations were associated with elevated blood pressure and a higher prevalence of preeclampsia [17], a major contributing factor for SGA births. Conversely, neonates with higher s(P)RR concentrations in cord blood (i.e. neonatal blood) typically exhibited a lower SGA birth prevalence. The present study also found that the association between s(P)RR concentrations in cord blood and SGA birth likelihood was independent of hypertensive disorders in pregnancy. Furthermore, our measurements of maternal s(P)RR concentrations at delivery identified no relationship between maternal s(P)RR concentration and s(P)RR concentration in cord blood (correlation coefficient 0.42). Given that we found no association between maternal s(P)RR concentration at delivery and the prevalence of SGA (data not shown), we believe that s(P)RR concentrations in cord blood are not significantly influenced by maternal s(P)RR at SGA births.

Furthermore, as SGA prevalence was significantly lower only in the fourth quartile group and showed no significant differences between the first, second and third quartile groups, we wonder if the association between s(P)RR concentrations in cord blood and SGA births applies only under a certain threshold.

Several studies suggest that SGA increases the risk for neurodevelopmental delay [14]–[15] and adult diseases, such as cardiovascular disease [13] and metabolic syndrome [24]–[27]. As recent evidence implies that (P)RR contributes to increased insulin resistance [28] and brain development [29], in addition to cardiovascular disease [13], [18], (P)RR may also be involved in adult diseases and neurodevelopmental delay in postnatal stages. This will need to be addressed in future studies.

Our study has some limitations worth nothing. First, we could not determine whether decreased s(P)RR concentration in fetal blood induced abnormal fetal body development or abnormal fetal development decreased s(P)RR levels because we assessed this association at only one point (i.e., only at birth). Assessment of s(P)RR concentration in fetal blood during pregnancy would clarify the contribution of (P)RR to fetal growth, although such a study may not be feasible. Second, the origin of s(P)RR in cord blood could not be determined, since the mechanism of placental transfer of s(P)RR from maternal circulation remains unclear. However, given that the molecular weight of s(P)RR is 28,000 [30], it appears to be too large to pass through the placenta. Indeed, previous studies have demonstrated that molecules larger than 1000 molecular weight cannot pass from maternal circulation to fetal circulation [31]. Third, our study population involved only Japanese people, so generalizability to other ethnic groups is limited. Finally, SGA prevalence in our study population was 8.9%, which was low compared to the typical SGA prevalence of approximately 10%, given its diagnostic criteria. This may be due to selection bias, as we selected only singleton neonates who lacked structural anomalies.

In conclusion, our study showed that higher s(P)RR concentration was associated with appropriate intrauterine fetal growth (i.e., less likely to be an SGA birth). Conversely, neonates with lower s(P)RR concentrations in cord blood may be abnormal in their intrauterine growth and represent follow-up candidates for adult diseases and neurodevelopmental disorders. The association between (P)RR and fetal origin of adult disease is an exciting and interesting theme for future investigation. Large, long-term cohort studies of (P)RR are needed to clarify this association.
